# Long-Term Effects of Dietary Protein and Branched-Chain Amino Acids on Metabolism and Inflammation in Mice

**DOI:** 10.3390/nu10070918

**Published:** 2018-07-18

**Authors:** Wei-Chieh Mu, Erin VanHoosier, Carrie M. Elks, Ryan W. Grant

**Affiliations:** 1 Department of Nutrition Science, Purdue University, West Lafayette, IN 47907, USA; h27352033@livemail.tw (W.-C.M.); evanhoosier@gmail.com (E.V.); 2 Matrix Biology Laboratory, Pennington Biomedical Research Center, Baton Rouge, LA 70808, USA; carrie.elks@pbrc.edu

**Keywords:** low protein diet, branched-chain amino acids, body composition, glucose homeostasis, inflammation

## Abstract

Aging is the main factor involved in the onset of degenerative diseases. Dietary protein restriction has been shown to increase the lifespan of rodents and improve metabolic phenotype. Branched-chain amino acids (BCAA) can act as nutrient signals that increase the lifespan of mice after prolonged supplementation. It remains unclear whether the combination of protein restriction and BCAA supplementation improves metabolic and immunological profiles during aging. Here, we investigated how dietary protein levels and BCAA supplementation impact metabolism and immune profile during a 12-month intervention in adult male C57BL/6J mice. We found that protein restriction improved insulin tolerance and increased hepatic fibroblast growth factor 21 mRNA, circulating interleukin (IL)-5 concentration, and thermogenic uncoupling protein 1 in subcutaneous white fat. Surprisingly, BCAA supplementation conditionally increased body weight, lean mass, and fat mass, and deteriorated insulin intolerance during protein restriction, but not during protein sufficiency. BCAA also induced pro-inflammatory gene expression in visceral adipose tissue under both normal and low protein conditions. These results suggest that dietary protein levels and BCAA supplementation coordinate a complex regulation of metabolism and tissue inflammation during prolonged feeding.

## 1. Introduction

With the advance of medicine and public health, the average human lifespan has increased. The proportion of elderly individuals who are 60–80 years old around the world was 12.3% in 2015 and is expected to exceed 20% by 2050 [1]. Although life expectancy has increased, there is little evidence of any concurrent increase in the period of time that individuals are healthy [2]. A broad spectrum of diseases occur during aging, such as cancer, cardiovascular disease, type 2 diabetes, osteoporosis, and Alzheimer’s disease [3]. Delay or prevention of the onset of age-associated morbidity and disease is critical to healthy aging in humans.

Degeneration of several normal functions has been reported with aging, including alterations in body composition (sarcopenic obesity) and declines in immune function. Sarcopenic obesity is characterized by both an excessive gain of body fat and a loss of muscle and bone mass during aging in the elderly [4,5]. Visceral adiposity leads to increased production of the pro-inflammatory mediators, such as tumor necrosis factor-α (TNF-α and monocyte chemoattractant protein-1 (MCP-1), by adipose tissue, which creates a pro-inflammatory microenvironment that results in immune cell infiltration and adipose tissue inflammation in mice and humans [6,7,8]. Additional hallmarks of aging include a decline in adaptive immunity and an increase in chronic inflammation, which are known as immunosenescence and inflamm-aging, respectively [9]. Age-related immunosenescence is largely due to thymic involution and the inability of aged naïve T cells to generate proper T-cell memory responses [10,11,12]. Immunosenescence negatively impacts vaccination efficacy especially in patients age 65 and older [13,14]. Inflamm-aging involves elevated pro-inflammatory IL-6, TNF-α, and acute phase proteins in the circulation during aging in humans [15,16,17,18,19]. Local inflammation in the adipose tissue and liver is an important source of pro-inflammatory cytokines, including IL-1β IL-6, and TNF-α that contributes to low-grade inflammation during aging in C57BL/6 mice [20,21,22,23,24]. The tissue and whole-body inflammation result in local or systemic insulin resistance due to the interference with insulin signaling by pro-inflammatory mediators [25]. Overall, these physiological alterations are associated with the development of age-related diseases [26,27].

Nutritional intervention is one potential approach to ameliorate age-associated dysfunction. Manipulating dietary macronutrient composition may be an alternative approach to mimicking the health benefits observed with caloric restriction in mice [28]. Dietary macronutrient composition has been shown to regulate the metabolism and longevity of C57BL/6 male mice [28,29,30]. In one study, diets low in protein and high in carbohydrates led to the longest median lifespan in ad libitum-fed C57BL/6 mice with metabolic benefits such as lower body weight and improved glucose tolerance [28]. Protein restriction induces metabolic changes including increased energy expenditure and food intake, decreased fat mass gain, and loss of lean mass in C57BL/6 mice [31,32]. Fibroblast growth factor 21 (FGF21) seems to be the endocrine signal of protein restriction that drives these metabolic changes [30,31,33]. Hepatic FGF21 is required for low protein diet-induced weight loss and thermogenic genes expression in subcutaneous white adipose tissue [34]. 

In addition to dietary protein ratio, a group of essential amino acids, referred to as branched-chain amino acids (BCAA), consisting of leucine, isoleucine, and valine, has been shown to play a role in lifespan extension in mice when supplemented with a BCAA-enriched amino acid mixture [35]. Research suggests that BCAA supplementation improves body composition and glucose metabolism and ameliorates hepatic and adipose tissue inflammation in mice [36,37,38,39,40]. Protein restriction and BCAA supplementation are suggested to be two potential dietary approaches to promote healthy aging. However, it is unknown whether the combination of a low protein diet and BCAA supplementation can improve metabolic and immunological profiles during aging. 

In this study, we investigated the role of dietary protein and a BCAA-supplemented diet on metabolic and immunological profiles of aging mice. We used a 2 × 2 factorial designed dietary intervention to assess how different levels of dietary protein and BCAA influence body composition, glucose homeostasis, and the aging immune system of adult male C57BL/6J mice. 

## 2. Materials and Methods

### 2.1. Animal Study

A 2 × 2 factorial design was used to determine how dietary protein levels and BCAA supplementation impact metabolic function and inflammatory profiles during aging. Animals were fed one of four test diets described in Table 1 (Research Diets, New Brunswick, NJ, USA). Dietary casein was included at the normal AIN-93M level (control diet) or half of the normal level (low protein diet). BCAA diets were supplemented with 11.1 g of leucine, 8.2 g of isoleucine, and 8.2 g of valine, which reflects BCAA amounts normally present in AIN-93M diet. Cornstarch was used to replace the energy of protein in the low protein diets. 

Male C57BL/6J mice were purchased from Jackson Laboratory (Bar Harbor, ME, USA) at 6-weeks old and maintained on chow diet (2018S, Teklad, Madison, WI, USA) before being randomized to one of the four diets at 4-months of age (*n* = 20–22 per group. Mice were sacrificed after 12 months of dietary intervention. A cohort of 4-month old C57BL/6J male mice (*n* = 28) fed the AIN-93M diet for 1 month was included as a young control reference. Mice were group housed at 22–24 °C on a 12 h light/dark cycle with free access to food and water. Body weight and food intake were recorded weekly. Food intake was measured by cage and normalized by the number of animals per cage. Animal experiments were approved by the Purdue Animal Care and Use Committee.

Mice were euthanized with carbon dioxide and blood samples collected via cardiac puncture into lithium heparin-coated tubes (365965, BD, San Jose, CA, USA). Plasma was collected after two 15-min 3000× *g* centrifugation steps, and stored at −80 °C until analysis. Tissues were collected and immediately frozen in liquid nitrogen.

### 2.2. Body Composition and Body Length Measurements

Body composition was determined at baseline, 6 months, and 12 months of dietary intervention. Briefly, mice were anesthetized under oxygen and isoflurane gas, then placed on a Lunar PIXImus dual energy X-ray absorptiometer (DXA; GE Medical Systems, Madison, WI, USA) to record body composition (lean mass, fat mass, and total tissue mass) and bone mineral density. Body length was measured at 12 months while mice were anesthetized. 

### 2.3. Femur Length Measurement

Femora were dissected from mice at sacrifice, attached soft tissues carefully removed, fixed in neutral buffered formalin (10% formaldehyde (F8775, Sigma-Aldrich, St. Louis, MO, USA), 4 g/L NaH2PO4 (S369, Thermo Fisher Scientific, Waltham, MA, USA), and 6.5 g/L Na2HPO4 (S374, Thermo Fisher Scientific, Waltham, MA, USA) in distilled water), and stored at 4 °C. Femur length was measured by a digimatic caliper (500-171-20, Mitutoyo Corporation, Kawasaki, Japan) with 0.01 mm precision.

### 2.4. Insulin and Glucose Tolerance Tests

Insulin tolerance tests (ITT) and glucose tolerance tests (GTT) were performed after 9 months of dietary intervention, when mice were 13 months of age, to examine systemic glucose homeostasis. We also performed ITT and GTT on the 4-month-old young reference group after 2 weeks of AIN-93M diet feeding, which allowed us to assess the effect of age on glucose homeostasis.

Prior to ITT and GTT mice were food-deprived for 4 or 12 h, respectively. Insulin (0.75 mU /g body weight, I0908, Sigma-Aldrich, St. Louis, MO, USA) or glucose (0.4 mg dextrose/g body weight, D9434, Sigma-Aldrich, St. Louis, MO, USA) solutions were administrated through intraperitoneal injection. Tail vein blood glucose levels were determined by glucometer (Breeze2, Bayer, Whippany, NJ, USA) at 0, 30, 60, 90, and 120 min after insulin or glucose administration. Due to the limited blood volume of mice, we did not collect blood samples to measure circulating insulin levels during the tests. 

### 2.5. Flow Cytometry

For isolation of thymocytes and splenocytes, thymus and spleen were freshly weighed and homogenized through 100-μm strainers (352360, Corning, Oneonta, NY, USA). Splenocytes and thymocytes were collected into RMPI 1640 media (11875-093, Gibco, Waltham, MA, USA) with 10% fetal bovine serum (FB-11, Omega Scientific, Tarzana, CA, USA), and 1% antibiotics (15140-122, Gibco, Waltham, MA, USA). The red blood cells within the thymocyte or splenocyte suspensions were lysed with ACK lysing buffer (118-156-101, VWR, Radnor, PA, USA) for 2 min and the reaction terminated with a 2X volume of media. Isolated cells were then filtered through a 40-μm strainer (352340, Corning, Oneonta, NY, USA) and counted using a hemacytometer; 4 million cells were used for staining. Zombie UV (423113, Biolegend, San Diego, CA, USA) was used to determine cell viability, and anti-CD16/CD32 antibody (eBioscience, 14-0161-85) was used to block Fc receptors and reduce non-specific binding. The fluorophore-conjugated antibodies for the thymic and splenic panels are below. For thymic flow cytometry analysis, we used anti-CD4 antibody (APC, 100412, Biolegend, San Diego, CA anti-CD8 antibody (BV510, 100752, Biolegend, San Diego, CA, USA), anti-CD44 antibody (FITC, 103040, Biolegend, San Diego, CA, USA), and anti-CD25 antibody (BV421, 101908, Biolegend, San Diego, CA, USA). For splenic flow cytometry analysis, we used anti-CD4 antibody (APC, 100412, Biolegend, San Diego, CA, USA), anti-CD8 antibody (BV510, 100752, Biolegend, San Diego, CA, USA), anti-CD44 antibody (FITC, 103040, Biolegend, San Diego, CA, USA), and anti-CD62L antibody (BV421, 104406, Biolegend, San Diego, CA, USA). Cells were stained in cell staining buffer (554656, BD, San Jose, CA, USA) for 30 min at 4 °C. The stained samples were analyzed with the LSRFortessa™ X-20 cell analyzer (BD, San Jose, CA, USA). FCS Express 6 software (De Novo Software, Glendale, CA, USA) was used to analyze cell populations.

### 2.6. Tissue RNA Isolation and Gene Expression Analysis

Total RNA of inguinal and epididymal white adipose tissues and liver were isolated with Trizol reagent (15596018, Thermo Fisher Scientific, Waltham, MA, USA) and Direct-zol RNA MiniPrep kit (R2052, Zymo Research, Irvine, CA, USA). RNA concentration and purity were assessed before cDNA synthesis using a NanoDrop 2000 Spectrophotometer (Thermo Fisher Scientific, Waltham, MA, USA). cDNA synthesis was performed using a High-Capacity cDNA Reverse Transcription Kit (ThermoFisher Scientific, 4368813). Gene expression levels were determined using quantitative real-time PCR with PrimeTime qPCR Assays (Table 2; Integrated DNA Technologies, Skokie, IL, USA) and TaqMan Gene Expression Master Mix (4369016, Thermo Fisher Scientific, Waltham, MA, USA) in the StepOnePlus Real-Time PCR System (Applied Biosystems, Foster City, CA, USA). Relative gene expression levels were normalized to Peptidyl-prolyl cis-trans isomerase *B* and calculated by 2^-delta Ct (threshold cycle)^ method.

### 2.7. Liver Proteomics

Liver samples were prepared using a previously described protocol [41]. Samples were analyzed using the Dionex UltiMate 3000 RSLC Nano System coupled to the Q Exactive™ HF Hybrid Quadrupole-Orbitrap MS (Thermo Fisher Scientific, Waltham, MA, USA). After digestion, peptides were loaded onto a trap column (20 µm × 350 mm) and washed using a flow rate of 5 µL/minute with 98% purified water/2% acetonitrile/0.01% formic acid. After 5 min, the trap column was switched in-line with a reverse phase Acclaim PepMap RSLC C18 (75 µm × 15 cm) analytical column. Peptides were separated on the analytical column using a 120-min method at a flow rate of 300 nL/minute. Mobile phase A consisted of 0.01% formic acid in purified water and a mobile phase B consisted of 0.01% formic acid in 80% acetonitrile. The linear gradient started at 5% B and reached 30% B in 80 min, 45% B in 91 min, and 100% B in 93 min. The column was held at 100% B for the next 5 min before being equilibrated at 5% B for 20 min. Samples were injected into the QE HF through the Nanospray Flex Ion Source fitted with a stainless-steel emission tip from Thermo Fisher Scientific. Data acquisition was performed monitoring the top 20 precursors at 120,000 resolution with an injection time of 100 milliseconds.

Acquired LC-MS/MS data files were searched against a mouse protein database downloaded from UniProtKB using Mascot Daemon version 2.5.1 (Matrix Science Inc., Boston, MA, USA). Data was searched using a precursor mass tolerance of 0.05 Da, an MS/MS mass tolerance of 0.2 Da, and one maximum missed cleavage. A fixed modification of ethanolyl on cysteine and oxidation of methionine as a variable modification were selected to be searched. To control the false discovery rate (FDR), a decoy search was performed, and peptide matches were accepted if the significance scores of their match had a *p* value < 0.05. The search results yielded FDR’s below 2%. A script was used to generate spectral counts from the exported peptide lists. Proteins were quantified using the spectral counts, and these spectral counts data were used for further downstream analysis.

A pre-filtered list of differentially expressed proteins (*p* < 0.05) between control and low protein groups was used for overrepresentation analysis (ORA), in which the enrichment of the number of proteins from a pathway list was compared to the total protein number in the pathway. Unmapped proteins were excluded from additional analysis. The ORA was conducted using the core analysis platform of the Ingenuity Pathway Analysis (IPA) software (Qiagen Bioinformatics; Redwood City, CA, USA), which identified overrepresented biological pathways with a statistically significant number of differentially expressed proteins between the control and low protein groups. Log_2_ ratios ≥1.0 and/or ≤−1.0 and *p* values of 0.05 were used as cutoff values in the analysis. 

### 2.8. Plasma Cytokine Analysis

Plasma pro-/anti-inflammatory cytokine levels were determined using the V-PLEX Pro-inflammatory Panel (K15048D, Meso Scale Discovery (MSD), Rockville, MD, USA) according to the manufacturer’s instructions. Briefly, the MSD plate was washed for three times with washing buffer (DPBS (D5652, St. Louis, MO, USA with 0.05% Tween-20 (BP337, Thermo Fisher Scientific, Waltham, MA, USA)). Calibrators and plasma samples in duplicates were added to the MSD plate and incubates overnight at 4 °C with 750 rpm shaking. The plate was washed three times, the detection antibody mixture added, and incubated for 2 h at room temperature with shaking at 750 rpm. The plate was washed three times and the read buffer added. The plate was read with the MESO QuickPlex SQ 120 (Meso Scale Discovery, Rockville, MD, USA).

### 2.9. Plasma FGF21 Concentration

Plasma FGF21 was determined using Rat/Mouse FGF21 ELISA Kit (EZRMFGF21-26K, EMD Millipore, Burlington, MA, USA) according to the manufacturer’s instructions. Briefly, the plate was washed three times. Calibrators, quality controls, and plasma samples in duplicates were added to the plate and mixed with detection antibody. The plate was incubated for 2 h at room temperature with shaking at 500 rpm. The plate was washed three times, enzyme solution added, and incubated for 30 min at room temperature with shaking. Substrate was added after three washes, and stop solution was added after 5 min of incubation with substrate. Absorbances were recorded at 450 nm and 590 nm by a microplate reader (Synergy H1 Hybrid Reader, BioTek, Winooski, VT, USA). Dose-response standard curves were fitted by four-parameter logistic functions. FGF21 concentrations were calculated using absorbance difference between 450 nm and 590 nm.

### 2.10. Statistical Analysis

Data represent individual values or mean ± standard error of the mean (SEM). Data were analyzed by two-way analysis of variance (ANOVA) or two-way ANOVA with repeated measures as indicated in the figure legends, and Tukey multiple comparisons for the post hoc test. The young and old control groups were compared by unpaired, two-tailed Student’s *t* test. Some data were transformed to meet the statistical assumptions for the residuals. A *p* value < 0.05 was considered to be statistically significant. ANOVA analysis was performed using SAS 9.4 software (SAS, Cary, NC, USA) and Student’s *t* test was performed using Microsoft Excel.

## 3. Results

### 3.1. BCAA Supplementation Conditionally Increased Body Weight, Lean Mass, and Fat Mass of Mice Fed a Low Protein Diet

**Figure 1:**
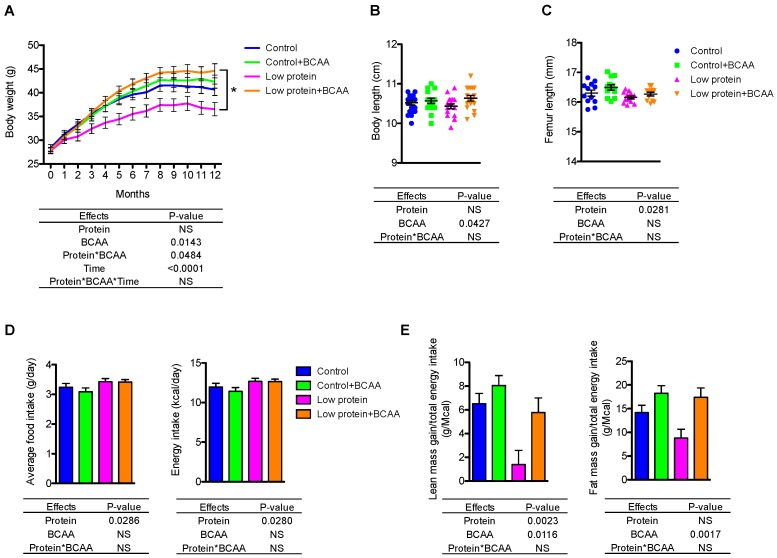
BCAA supplementation conditionally increased body weight in mice fed a low protein diet. Body weight (**A**), body length (**B**), and femur length. (**C**) were measured (*n* = 12–20 mice/group). The average daily food intake (g/cage/day normalized by the number of mice per cage) and energy intake (kcal/cage/day normalized by the number of mice per cage) (**D**) and lean and fat mass gain normalized to energy intake (**E**) were also calculated (*n* = 6–7 cages/group). Data represent mean ± standard error of the mean (SEM). Statistical analyses of main effects (protein, BCAA, and time) and interactions (protein*BCAA and protein*BCAA*time) are shown below each graph. * *p* < 0.05 for low protein vs. low protein + BCAA in panel A. BCAA: Branched-chain amino acids.

Due to the long-term nature of the study, 10 mice died prior to study endpoint (control *n* = 2; control + BCAA *n* = 3; low protein *n* = 2; low protein + BCAA *n* = 3) from causes unrelated to treatment leaving final group numbers at 17–20 mice/group. The low protein group had significantly lower body weight than the low protein + BCAA group due to a significant interaction effect of dietary protein levels and BCAA supplementation (*p* = 0.0484) (Figure 1A). BCAA supplementation significantly increased (*p* = 0.0427) the nose-to-tail body length after 12-month dietary intervention (Figure 1B). We also measured femoral length after the intervention as an indicator of growth; the low protein diet decreased femoral length (*p* = 0.0281), while BCAA supplementation showed a trend toward increasing femoral length (*p* = 0.0684) (Figure 1C).

To understand how diet influences the distribution of lean and fat mass, we monitored the body composition at baseline, 6 months, and 12 months using DXA imaging. Not surprisingly, the low protein group had significantly lower lean mass compared to the other three groups at 12 months due to a significant three-way interaction effect of dietary protein levels, BCAA supplementation, and time (month) (*p* = 0.0483) (Table 3 and Table S1). Interestingly, BCAA supplementation increased lean mass only under the low dietary protein condition and showed no further enhancement under normal protein condition (Table 3). BCAA supplementation increased lean mass gain during protein restriction to levels similar to those seen in mice on the control diet. Further, mice continuously accrued fat mass throughout the 12-month intervention (Table 3). The low protein group had lower fat mass compared to the low protein + BCAA group due to a 3-way interaction (*p* = 0.0245) (Table 3 and Table S1). These data suggest that dietary protein levels and BCAA supplementation impact both lean and fat mass after long-term feeding.

Despite having decreased lean and fat mass gain, mice consuming the low protein diets had increased average daily food (*p* = 0.0286) and energy intake (*p* = 0.0280) (Figure 1D). The hyperphagic effect of low protein diets has been reported previously in rodents [28,31,32,42]. We normalized the lean or fat mass gain to total energy intake, which allowed us to evaluate energy partitioning into lean mass or fat mass. Low protein diet significantly reduced lean mass gain (*p* = 0.0023), and marginally decreased the energy stored as fat mass (*p* = 0.0911) (Figure 1E). Conversely, BCAA supplementation enhanced both lean mass gain (*p* = 0.0116) and fat mass gain (*p* = 0.0017) when normalized to total energy intake (Figure 1E). These data suggest that dietary protein levels play a role in determining lean mass, while BCAA supplementation promotes energy storage in both lean and fat tissues.

### 3.2. Dietary Protein Had an Impact on Bone Mineral Density

Femoral bone mineral density (fBMD) increased over time (*p* < 0.05, Table 3). The largest increases were seen between baseline and 6 months. For fBMD, there was an interaction between BCAA and dietary protein (*p* < 0.05) driven by the low-protein + BCAA group, which did not change over time. Femoral bone mineral content (fBMC) significantly decreased over time; however, there were no differences between protein, BCAAs, and time. There was a trend (*p* = 0.0571) for the low protein groups to have lower fBMC. 

### 3.3. The Low Protein Group Exhibited Improved Insulin Tolerance after 9-Month Dietary Intervention

**Figure 2:**
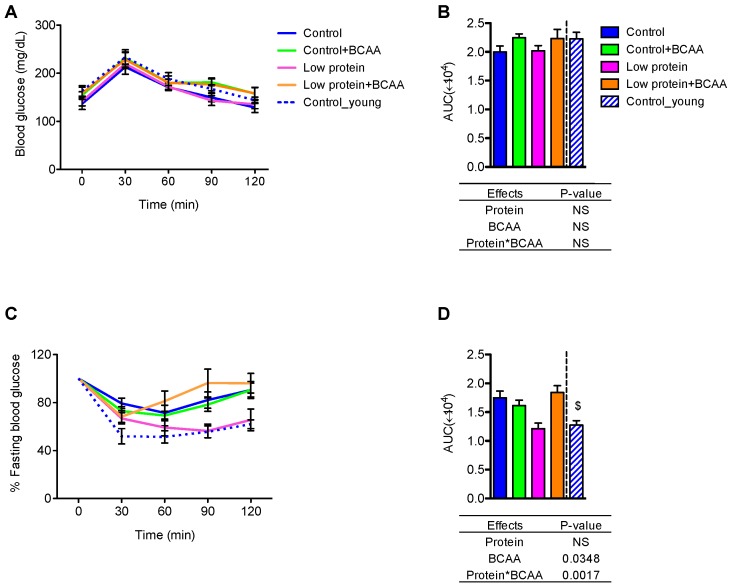
The low protein diet improved insulin tolerance but did not affect glucose tolerance. Blood glucose across time (**A**) and area under the curve (AUC) (**B**) were recorded calculated from the GTT. The response to insulin as a percentage of fasting blood glucose was calculated for the ITT time course (**C**) and the corresponding AUC (**D**). Data represent mean ± SEM (*n* = 8–12 mice/group). Statistical analyses of main effects (protein and BCAA) and interactions (protein*BCAA) are shown below panels (**B**,**D**). $ *p* < 0.05 between the young and the old control groups by Student’s *t* test. GTT: glucose tolerance test; ITT: Insulin tolerance test.

To study the effects of dietary protein and BCAA on glucose homeostasis, we performed GTT and ITT after 9 months of dietary intervention. No differences between treatment or age groups were observed in the average blood glucose values over time (Figure 2A). However, BCAA supplementation marginally increased area under the curve (AUC) (*p* = 0.0695) (Figure 2B). All mice appeared to respond similarly to exogenous glucose administration. 

During the ITT, the low protein group displayed similar patterns in blood glucose change over time and AUC during the ITT as the young control group (Figure 2C,D). The low protein group also exhibited improved insulin tolerance when compared to the control or low protein + BCAA groups, as demonstrated by reduced AUC (Figure 2D). Our data also show an age effect on insulin tolerance, with decreased AUC of the young control group compared to the control group (*p* < 0.05) (Figure 2D). These results indicate that prolonged low protein diet feeding leads to better insulin tolerance in aging mice.

### 3.4. Low Protein Diet Upregulated Fgf21 mRNA in Liver and Thermogenic Gene Expression in Inguinal White Adipose Tissue

**Figure 3:**
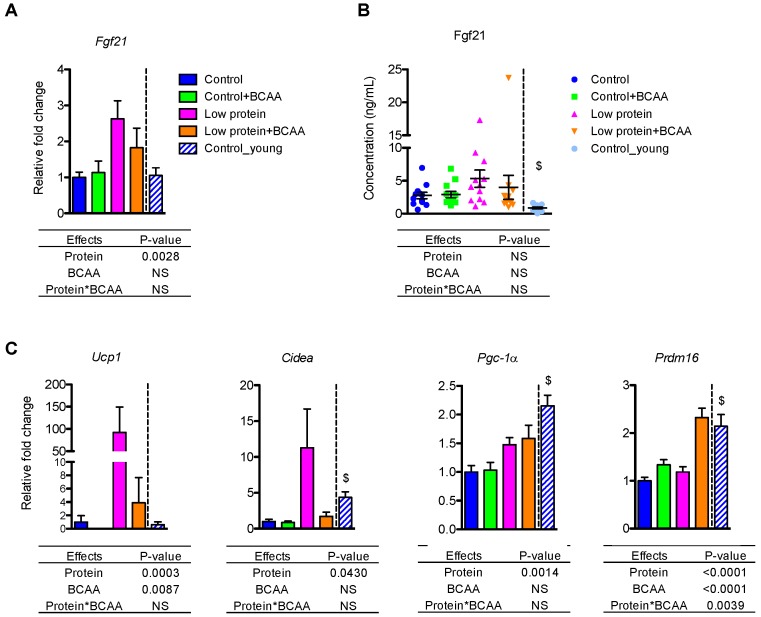
Low protein diet upregulated Fgf21 mRNA in the liver and thermogenic gene expression in inguinal white adipose tissue (IWAT). The liver mRNA expression of Fgf21 (**A**) and plasma FGF21 concentrations (**B**) were measured. The mRNA expression of Ucp1, Cidea, Pgc-1α, and Prdm16 was measured in IWAT (**C**). Data represent mean ± SEM (*n* = 8–12 mice/group). Statistical analyses of main effects (protein and BCAA) and interactions (protein*BCAA) are shown below each graph. $ *p* < 0.05 for young vs. old control groups.

FGF21 is a hormonal signal required for the metabolic adaptations, such as browning of white adipose tissue, during protein restriction in mice [32,34]. The low protein diet upregulated hepatic Fgf21 mRNA levels (*p* = 0.0028) (Figure 3A). Although there was no effect of treatment on circulating FGF21 levels due to large variation within groups, the pattern of circulating FGF21 concentrations was similar to that of the hepatic Fgf21 mRNA levels (Figure 3B). 

Because dietary protein restriction can upregulate thermogenic gene mRNA expression through Fgf21 [31,32], we examined thermogenic gene expression in inguinal white adipose tissue (IWAT). Low protein diet markedly induced Ucp1 mRNA expression (*p* = 0.0003), but BCAA supplementation significantly reduced Ucp1 mRNA (*p* = 0.0087) (Figure 3C). Low protein diet also increased Cidea (*p* = 0.043) and Pgc-1α (*p* = 0.0014) mRNA levels (Figure 3C). The low protein + BCAA group had higher *Prdm16* mRNA level compared to the other three groups due to an interaction of dietary protein levels and BCAA supplementation (*p* = 0.0039) (Figure 3C). These results suggest that chronic protein restriction could stimulate browning of white adipose tissue through the upregulation of key thermogenic genes.

### 3.5. BCAA Supplementation Induced Pro-Inflammatory Gene Expression in White Adipose Tissue and Liver

**Figure 4:**
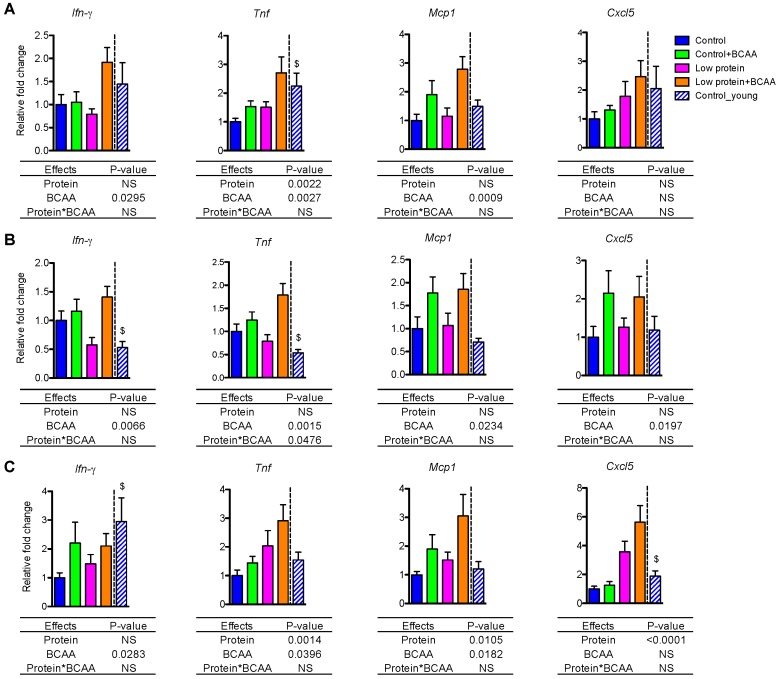
BCAA supplementation induced Ifn-γ, Tnf, and Mcp1 expression in white adipose tissue and liver, while Cxcl5 expression was differentially regulated by diets across tissues. The relative gene expression levels of pro-inflammatory Ifn-γ, Tnf, Mcp1, and Cxcl5 were measured in IWAT (**A**), epididymal white adipose tissue (EWAT) (**B**), and liver (**C**). Data represent mean ± SEM (*n* = 8–12 mice per group). Statistical analyses of main effects (protein and BCAA) and interactions (protein*BCAA) are shown below each graph. $ *p* < 0.05 for young vs. old control groups.

Age-associated low-grade, chronic inflammation in metabolic tissues may contribute to insulin resistance [43,44]. We examined pro-inflammatory cytokine and chemokine mRNA levels in IWAT, epididymal white adipose tissue (EWAT), and liver. BCAA supplementation significantly upregulated mRNA expression of proinflammatory cytokines Ifn*-γ*, *Tnf*, and chemokine Mcp1 in IWAT, EWAT, and liver (all *p* < 0.05, Figure 4A–C). BCAA supplementation also induced Cxcl5 mRNA (*p* = 0.0197) in EWAT (Figure 4B). Low protein diet significantly increased Tnf (*p* = 0.0022) and marginally enhanced Cxcl5 mRNA (*p* = 0.0615) in IWAT (Figure 4A). Low protein diet also enhanced Tnf (*p* = 0.0014), Mcp1 (*p* = 0.0105), and Cxcl5 (*p* < 0.0001) mRNA in liver (Figure 4C). In addition, Cxcl5 was differentially regulated by the dietary interventions in different tissues. Cxcl5 mRNA expression was enhanced by BCAA supplementation in EWAT (Figure 4B) but was increased by low protein diet in liver (Figure 4C).

Notably, there is a tendency for the interaction effect of dietary protein and BCAA to influence Ifn-γ mRNA in IWAT (*p* = 0.0507) and EWAT (*p* = 0.0610), which suggests that BCAA supplementation in the low protein diet induced a greater expression of Ifn-γ mRNA (Figure 4A,B). More importantly, for most of the genes reported here we can see higher expression levels in the low protein + BCAA group, which indicates that the combination of a low level of dietary protein with a high amount of BCAA is driving the pro-inflammatory gene expression in metabolic tissues. Collectively, these data suggest that the low protein + BCAA diet has strong potential for increasing the expression of pro-inflammatory genes in adipose tissue and liver. 

### 3.6. Dietary Intervention Differentially Impacted Anti-Inflammatory Gene Expression in White Adipose Tissue and Liver

**Figure 5:**
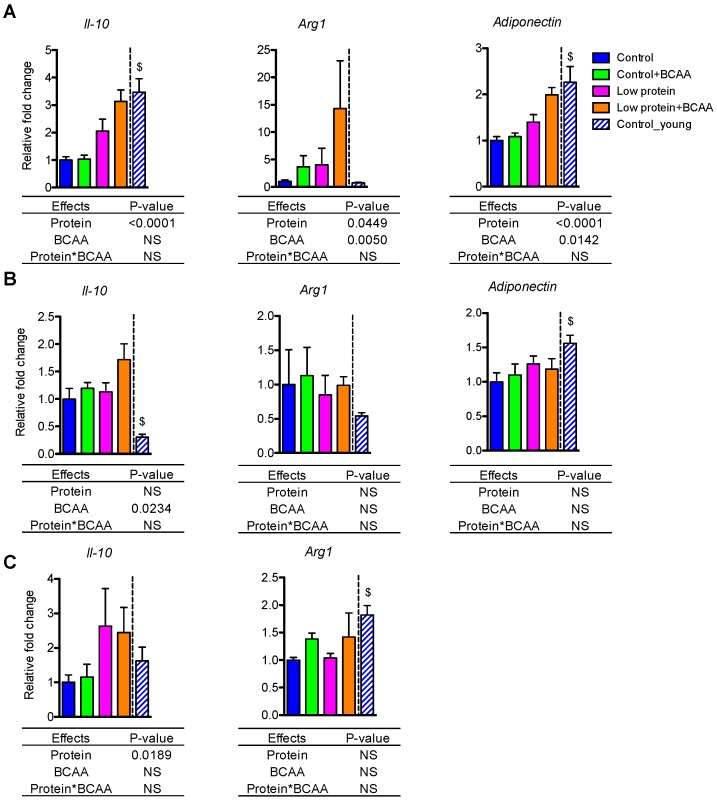
Dietary intervention differentially impacted anti-inflammatory gene expression in white adipose tissue and liver. The relative gene expression levels of anti-inflammatory Il-10, Arg1, and adiponectin were measured in IWAT (**A**) and EWAT (**B**) and liver (**C**). Data represent mean ± SEM (*n* = 8–12 mice/group). Statistical analyses of main effects (protein and BCAA) and interactions (protein*BCAA) are shown below each graph. $ *p* < 0.05 for young vs. old control groups.

The anti-inflammatory genes exhibited tissue-specific patterns in IWAT, EWAT, and liver. Low protein diet induced Il-10 mRNA expression in IWAT (*p* < 0.0001) and liver (*p* = 0.0189) (Figure 5A,C), while BCAA supplementation enhanced Il-10 mRNA expression in EWAT (*p* = 0.0234) (Figure 5B). Low protein diet and BCAA supplementation increased Arg1 (*p* = 0.0449, *p* = 0.005) and adiponectin (*p* < 0.0001, *p* = 0.0142) mRNA expression in IWAT (Figure 5A). Aging resulted in reduced Il-10 and adiponectin in IWAT (*p* < 0.05 for both), reduced adiponectin and increased Il-10 in EWAT (*p* < 0.05 for both), and reduced Arg1 in the liver (*p* < 0.05) (Figure 5A–C).

### 3.7. BCAA Supplementation Increased Thymus Weight, while the Dietary Intervention Had No Effects on Thymocyte Cell Counts or Different T-Cell Populations in Thymus

**Figure 6:**
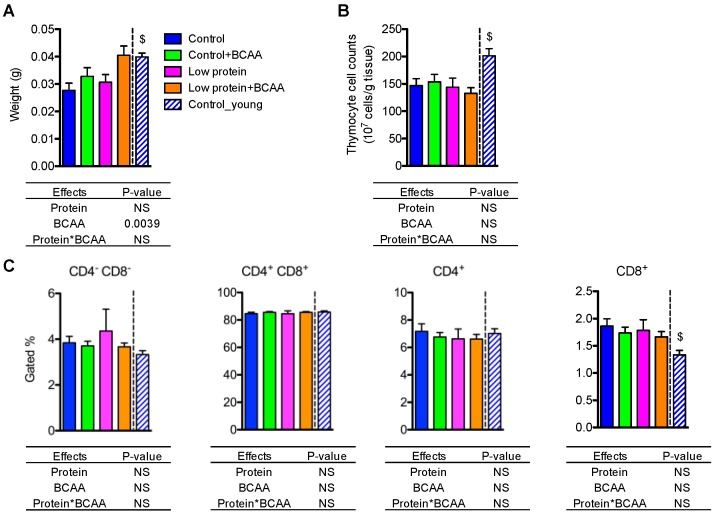
BCAA supplementation increased thymus weight but did not affect thymocyte cell counts or T-cell populations. Thymus weight was recorded at end of study (**A**), and thymocyte cell counts were calculated (**B**). The quantification of CD4^−^CD8^−^ double negative, CD4^+^CD8^+^ double positive, CD4^+^ single positive, and CD8^+^ single positive T-cell populations in the thymus (**C**). Data represent mean ± SEM (*n* = 11–12 mice/group). Statistical analyses of main effects (protein and BCAA) and interactions (protein*BCAA) are shown below each graph. $ *p* < 0.05 for the young vs. old control groups.

Thymic involution is a hallmark of aging [10]. We observed potent effects of age on thymus weight (*p* < 0.05) and thymocyte cell counts (*p* < 0.05), which were significantly lower in the old control group compared to the young control group (Figure 6A,B). BCAA supplementation significantly increased thymus weight (*p* = 0.0039), but dietary intervention had no impact on thymocyte cell counts (Figure 6A,B). These results suggest that BCAAs increase thymus weight, but neither BCAAs nor a low-protein diet rescues age associated changes in thymocyte cell density.

To investigate whether dietary interventions influenced the composition of different T-cell compartments during thymic T-cell maturation, we examined thymic CD4^−^CD8^−^ double negative, CD4^+^CD8^+^ double positive, CD4^+^ single positive, and CD8^+^ single positive T-cell subpopulations using flow cytometry (Figure S1). There were no differences in the percentages of gated cells of all T-cell subpopulations among the dietary interventions (Figure 6C). However, the young control group had lower percentages of CD8^+^ single positive T cells compared to the control group (*p* < 0.05) (Figure 6C). Collectively, these data suggest that the dietary intervention had no effect on the composition of different T-cell subpopulations in the thymus.

### 3.8. BCAA Supplementation Increased Spleen Weight, while Dietary Intervention Had No Impact on Splenocyte Cell Counts and the Percentages of the Naive and the Memory T Lymphocytes in Spleen

**Figure 7:**
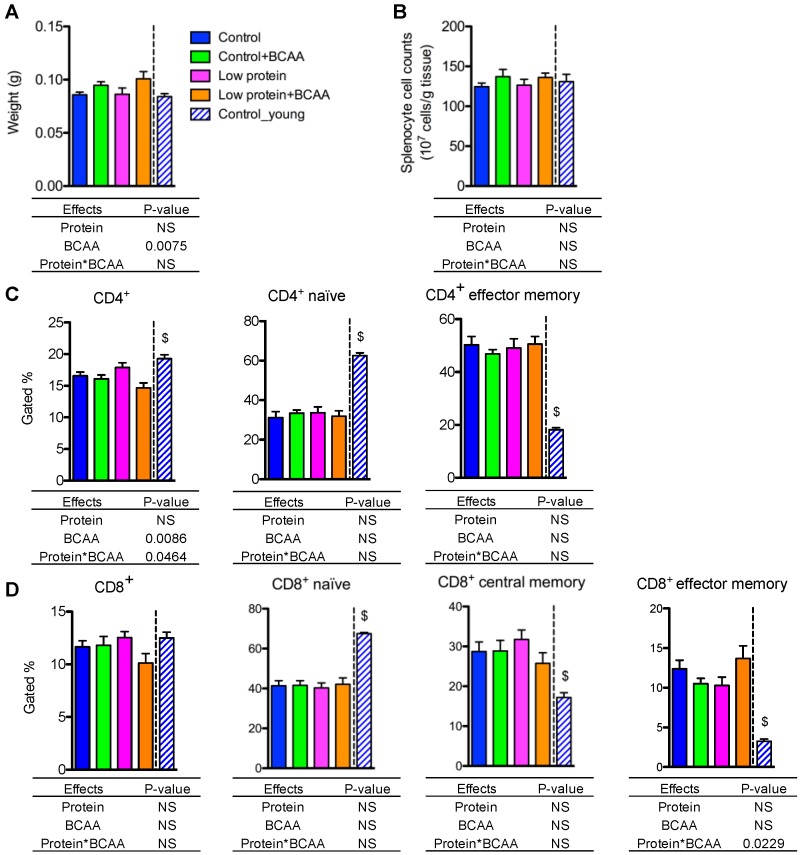
BCAA supplementation increased spleen weight, while dietary intervention had no impact on splenocyte cell counts and the percentages of naive and memory T lymphocytes in spleen. Spleen weight was recorded (**A**), and splenocyte cell counts were calculated (**B**). The quantification of CD4^+^, and the naïve (CD4^+^CD62L^+^CD44^−^) and memory (CD4^+^CD62L^−^CD44^+^) CD4^+^ subpopulations in spleen (**C**). The quantification of CD8^+^, and the naïve (CD8^+^CD62L^+^CD44^−^), central memory (CD8^+^CD62L^+^CD44^+^), and effector memory (CD8^+^CD62L^−^CD44^+^) CD8^+^ subpopulations in spleen (**D**). Data represent mean ± SEM (*n* = 11–12 mice/group). Statistical analyses of main effects (protein and BCAA) and interactions (protein*BCAA) are shown below each graph. $ *p* < 0.05 for the young vs. old control groups.

The spleen is the largest secondary lymphoid organ that contains CD4^+^ helper and CD8^+^ cytotoxic T lymphocytes [45]. BCAA supplementation significantly increased spleen weight (*p* = 0.0075), but splenocyte cell counts were unaffected by dietary intervention (Figure 7A,B). CD4^+^ T lymphocyte frequency was lower in the low protein + BCAA group when compared to the other three groups due to an interaction effect of dietary protein and BCAA (*p* = 0.0464) (Figure 5C and Figure S2A). CD8^+^ T cell frequency displayed a similar trend (*p* = 0.0587 for the interaction effect) to that of CD4^+^ T cells (Figure 7D and Figure S2A).

One signature of the aging immune system is an altered splenic naïve and memory T-cell ratio [46,47]. Aging decreases naïve T cells, but increases memory T cells, in peripheral lymphoid tissues [46,47]. The control group had markedly lower CD4^+^ and CD8^+^ naïve (CD44^−^CD62L^+^) T-cell populations, and significantly higher CD4^+^ and CD8^+^ memory (CD44^+^CD62L^−^) T-cell populations compared to the young control group (*p* < 0.05) (Figure 7C,D and Figure S2B,C). The percentages of CD4^+^ naïve (CD4^+^CD44^−^CD62L^+^) and CD4^+^ effector memory (CD4^+^CD44^+^CD62L^−^) T lymphocytes were not altered by dietary intervention (Figure 7C). Similarly, the frequencies of the CD8^+^ naïve (CD8^+^CD44^−^CD62L^+^), CD8^+^ central memory (CD8^+^CD44^+^CD62L^+^), and CD8^+^ effector memory (CD8^+^CD44^+^CD62L^−^) T cells were not affected by dietary intervention (Figure 7D). Even though we detected a significant interaction effect on the percentages of CD8^+^ effector memory T cells (*p* = 0.0229), the Tukey post hoc test revealed no differences among the dietary interventions (Figure 7D). These data indicate that aging is the most significant driver of the changes in naïve and memory T lymphocytes in spleen, while our dietary interventions had no effect on reversing the age-associated alterations of splenic naïve and memory T-cell populations.

### 3.9. Low Protein Diet Induced Circulating IL-5 after Prolonged Intervention

**Figure 8:**
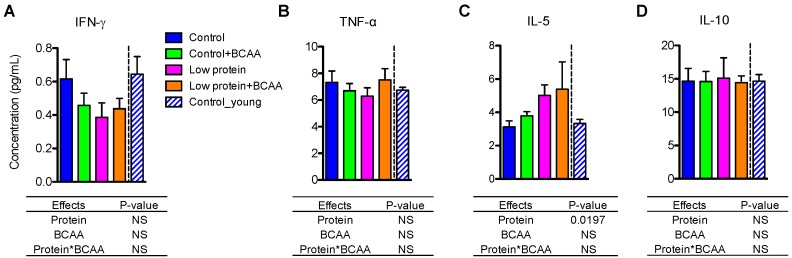
Low protein diet increased circulating IL-5 levels. Plasma pro-inflammatory cytokines IFN-γ (**A**) and TNF-α (**B**), and anti-inflammatory cytokines IL-5 (**C**) and IL-10 (**D**) were determined using multiplex assays. Data represent mean ± SEM (*n* = 12 mice/group). Statistical analyses of main effects (protein and BCAA) and interactions (protein*BCAA) are shown below each graph.

Unlike the tissue pro- and anti- inflammatory cytokine gene expression levels (Figure 4 and Figure 5), plasma IFN-γ, TNF-α, and IL-10 concentrations were unaffected by dietary interventions or age (Figure 8A,B,D). Interestingly, we found that circulating IL-5 levels were significantly increased by the low protein diet (*p* = 0.0197) (Figure 8C). 

### 3.10. Liver Proteomics

The top five overrepresented canonical pathways between the control and low protein groups included: oxidative phosphorylation, mitochondrial dysfunction, superpathway of citrulline metabolism, urea cycle, and citrulline biosynthesis (Table 4). The same analysis was also conducted to compare the low protein and low protein + BCAA groups, with differential regulation found in the following top 5 canonical pathways: tyrosine degradation I, urea cycle, cysteine biosynthesis/homocysteine degradation, superpathway of citrulline metabolism, and TCA cycle II (eukaryotic) (Table 4). Our Ingenuity Pathway Analysis also generated a priority list for top regulated biofunctions for the control vs. low protein and low protein vs. low protein + BCAA comparisons, using the principles of overrepresentation analysis as described above. Three of the top 5 biofunctions were identical for both comparison groups: amino acid metabolism, small molecule biochemistry, and cell signaling. The additional top biofunctions in the control vs. low protein comparison were nucleic acid metabolism and post-translational modification. The additional top biofunctions in the low protein vs. low protein + BCAA comparison were lipid metabolism and molecular transport.

## 4. Discussion

In this study, we examined the chronic effects of protein restriction and BCAA supplementation on metabolic and immunological profiles in adult male C57BL/6J mice. The low protein group had significantly lower lean mass compared to all other groups after 12 months of feeding. The low protein group also had lower body weight and fat mass compared to the low protein + BCAA group, which suggests that BCAA are more effective in modulating body composition during protein restriction. The low protein group exhibited improved insulin tolerance that was similar to that observed in young mice. As expected, thermogenic gene expression in subcutaneous white adipose tissue and Fgf21 mRNA in liver were upregulated by protein restriction. Tissue inflammatory gene expression profiles suggest a consistent induction of pro-inflammatory mRNA levels by BCAA in visceral white adipose tissue, especially under low protein conditions. These data imply that dietary protein levels and BCAA play a role in modulating whole-body metabolism as previously suggested [28,31,32].

Our findings are consistent with previous literature demonstrating that BCAA differentially impact body weight maintenance in response to different diets. We report that low protein diets moderately increased the daily average energy intake of mice, while BCAA supplementation did not affect energy intake. Moderate protein restriction has been reported to induce hyperphagia in mice and rats [28,31,32,42,48]. On the other hand, BCAA, especially leucine, is the amino acid signal that triggers anorexia when centrally administered to the hypothalamus of male Long-Evans rat [49]. Our data suggest that low dietary protein content is a stronger factor than BCAA supplementation in determining energy intake of male C57BL/6J mice. Even though the two protein-restricted groups had similar energy intakes, the body weight, lean mass, and fat mass of mice supplemented with BCAA were markedly higher compared to the non-supplemented group. These data imply that the low protein diet provided sufficient essential amino acids to support the formation of lean mass in the presence of BCAA supplementation. Further, long-term BCAA supplementation in the low-protein diet led to the accumulation of both lean and fat mass, but showed no impact when supplemented in the normal protein diet. These data indicate that male C57BL/6J mice may have different sensitivities to exogenous BCAA depending on the dietary protein levels. BCAA supplementation has been shown to differentially impact body weight according to dietary fat levels in C57BL/6J mice [50,51]. BCAA supplementation decreases body weight and fat mass of C57BL/6J mice in response to high-fat diets, but not to normal-fat diets [50,51]. Taken together, the sensitivity to BCAA supplementation is likely to vary based on the macronutrient composition of the diet.

Insulin tolerance and glucose tolerance are important indicators of whole-body glucose metabolism. We found that the low protein group had improved insulin tolerance compared to the control and the low protein + BCAA groups and shared a similar response with young control mice. These data again suggest an interaction of dietary protein and BCAA. In the low protein condition, BCAA exacerbated insulin intolerance, but this did not occur under the normal protein condition. We have identified three contributing factors to this improvement in insulin action by the low protein diet; namely, hepatic Fg21, white adipose tissue browning, and circulating IL-5. FGF21 is an endocrine hormone that is primarily produced by the liver during protein restriction in both rodents and humans [31,32,34]. FGF21 plays a role in improving glucose homeostasis through browning of the white adipose tissue in mice [52,53,54,55,56]. We found elevated Fgf21 mRNA expression in liver induced by the low protein diet; however, circulating FGF21 levels remained unchanged. These results imply that our protein restriction was effective enough to induce hepatic Fgf21 mRNA but was not as restrictive as those previously used to increase circulating Fgf21 in C57BL/6 mice [31,32,34]. Consistently, the thermogenic genes Ucp1, Cidea, and Pgc-1α in subcutaneous white fat were increased by protein restriction. White adipose tissue browning in mice is accompanied by an improvement in glucose tolerance and insulin sensitivity [57]. Solon-Biet et al. reported that C57BL/6 mice fed a diet low in protein and high in carbohydrate had better glucose tolerance with higher Ucp1 mRNA in brown adipose tissue, higher hepatic Fgf21 mRNA, and higher circulating FGF21 [28,30]. Lastly, we identified another metabolic regulator, circulating IL-5, to be elevated by chronic protein restriction. Transgenic overexpression of IL-5 in mice leads to improved glucose tolerance and reduced visceral adiposity compared to wild-type controls [58]. IL-5 is produced in adipose tissue by group 2 innate lymphoid cells which contribute to adipose tissue browning, making this pathway a candidate for the metabolic differences noted in this study. Future studies mechanistic studies examining the impact of IL-5 on protein restriction will be needed to determine the importance of this. In summary, our data indicate that a low protein diet may enhance insulin tolerance through stimulation of adaptive thermogenesis in subcutaneous white fat, and this may be linked to circulating IL-5. 

In addition to changes in IWAT thermogenic gene expression, proteomic analysis of the liver revealed impact on metabolic pathways. Compared to the control diet, the low protein diet upregulated proteins associated with oxidative phosphorylation and the urea cycle. It has been noted that a profile of upregulated fatty acid oxidation occurs in the liver during caloric restriction in mice and methionine restriction in rats [59,60]. During caloric restriction there is also upregulation of the urea cycle, which is likely related to increased TCA cycle anapleurosis in mice [60]. Our results indicate that similar responses may occur during a low protein diet. It is also interesting that the urea cycle changes were not mitigated by supplementation of the low protein diet with BCAAs, while some of the physiological changes (e.g., body weight loss) were attenuated.

We demonstrate a complex manipulation of inflammatory genes by dietary protein levels, BCAA supplementation and the interaction of the two factors. The low protein + BCAA diet seems to be a potential inducer of visceral adipose tissue inflammation in our study. In contrast to our findings, Terakura et al. reported that BCAA supplementation markedly ameliorated adipose tissue inflammation in *db/db* mice by decreasing Tnf-α, Il-6 and Mcp1 mRNA levels, and decreasing macrophage infiltration into gonadal white fat [40]. These results suggest that BCAA supplementation may be beneficial in ameliorating visceral adipose tissue in the settings of genetic obesity and diabetes, but not in normal and healthy conditions. In addition to its apparent inflammatory effects on visceral adipose tissue, we also show that BCAA supplementation enhanced Ifn-γ Tnf, and Mcp1 mRNA expressions in the inguinal white adipose tissue and the liver. Terakura et al. demonstrated that BCAA supplementation significantly reduced hepatic steatosis and suppressed inflammatory gene expression in the livers of *db/db* mice [40], while Zhang et al. found that BCAA supplementation aggravated the markers of liver damage in C57BL/6J mice caused by high-fat diet through mTOR activation [50]. These results again imply that BCAA may have opposing roles in modulating tissue inflammatory status in mice depending on genetic background and/or dietary conditions. On the other hand, we found that protein restriction significantly increased anti-inflammatory genes (Il-10, Arg-1) and Tnf mRNA levels in the inguinal fat. The accumulation of alternatively activated macrophages in the subcutaneous fat is essential for browning of white adipose tissue and can contribute to thermogenic responses [61]. We also found no differences of the circulating cytokine concentrations among groups including pro-inflammatory IFN-γ and TNF-α, and anti-inflammatory IL-10. These results indicate that protein restriction and BCAA supplementation affect local, but not systemic, inflammatory profiles in our animal model.

We demonstrate a significant age effect on thymus weight and splenic naïve and memory T-cell populations as expected [47]. Even though BCAA supplementation markedly increased thymus and spleen weights, it did not contribute to higher cell counts in the tissues. Diet-induced obesity promotes perithymic fat accumulation and reduces thymocyte cell counts in C57BL/6 mice after 13 months of feeding [62], and future research will be needed to determine the effect of BCAAs and dietary protein on perithymic fat accumulation. Additionally, our dietary intervention had no effects on thymic T-cell composition or splenic naïve and memory T-cell populations. Le Couteur et al. reported that aged C57BL/6 mice (15-month-old) with low protein intake had a rejuvenated T-cell profile in the spleen, including increased naïve T cells, and fewer memory T cells [29]. This inconsistency may be due to the different ages of treatment onset (4 weeks, immature mouse [29] versus 4 months mature mouse) and different protein composition of the test diets [29]. It is worth noting that they used dietary protein ranging from 5% to 60% of energy, but we used a narrow range from 7% to 16% [29]. Further, the degree of prevention for age related changes is difficult to evaluate in the absence of young control mice when examining splenic T-cell profiles [29]. Hence, it remains unclear if the rejuvenated T-cell profile induced by low protein intake in that study indeed represents the reversal of immunosenescence during aging. In summary, our data suggest that dietary protein and BCAA may not play a significant role in the aging spleen and thymus. 

## 5. Conclusions

Dietary protein and BCAA are essential components of the diet and normally necessary for the maintenance of normal organ and tissue function, and they are also commonly used as dietary supplements. Traditionally the role of protein in aging has been seen through the lens of sarcopenia, the loss of skeletal muscle mass and function, with the idea that greater protein intake is beneficial for maintaining muscle mass. However, our data and the data of others suggest complex interactions between amino acids and/or dietary protein on metabolism and inflammation and argue that the effects protein on other tissues and systems need to be considered. 

## Figures and Tables

**Table 1:** Diet ingredients.

Ingredient (g)	Control	Control + BCAA	Low Protein	Low Protein + BCAA
Casein	140	140	70	70
L-Leucine	0	11.1	0	11.1
L-Isoleucine	0	8.2	0	8.2
L-Valine	0	8.2	0	8.2
L-Cystine	1.8	1.8	1.8	1.8
Corn starch	496	468	544	516
Maltodextrin 10	125	125	125	125
Sucrose	100	100	100	100
Cellulose	50	50	50	50
Soybean oil	40	40	40	40
t-Butylhydroquinone	0.008	0.008	0.008	0.008
Mineral Mix S10022M	35	35	35	35
Vitamin Mix V10037	10	10	10	10
Choline Bitartrate	2.5	2.5	2.5	2.5
Macronutrient composition (kcal%)	Control	Control + BCAA	Low protein	Low protein + BCAA
Protein	13	16	7	10
Carbohydrate	77	74	83	80
Fat	10	10	10	10
kcal/g	3.7	3.7	3.8	3.8

BCAA: Branched-chain amino acids.

**Table 2:** Primer sequences.

Gene	Primer Sequence
Ppib	F AGCAAGTTCCATCGTGTCATC
	R CCGTAGTGCTTCAGCTTGA
Ifn-γ	F CTGAGACAATGAACGCTACACA
	R TCCACATCTATGCCACTTGAG
Mcp1	F CATCCACGTGTTGGCTCA
	R AACTACAGCTTCTTTGGGACA
Ucp1	F CAAATCAGCTTTGCCTCACTC
	R CACACCTCCAGTCATTAAGCC
Adiponectin	F TGTCTGTACGATTGTCAGTGG
	R GCAGGATTAAGAGGAACAGGAG
Pgc-1α	F AGAAGTCCCATACACAACCG
	R GGTCACTGGAAGATATGGCA
Cidea	F TCAAACCATGACCGAAGTAGC
	R GTAACCAGGCCAGTTGTGAT
Fgf21	F CAGCCTTAGTGTCTTCTCAGC
	R GGGATGGGTCAGGTTCAGA
Prdm16	F CACAAGACATCTGAGGACACA
	R CACTTGAACGGCTTCTCTTTG
Cxcl5	F TTCTGTTGCTGTTCACGCT
	R ATCACCTCCAAATTAGCGATCA
Arg1	F GAATGGAAGAGTCAGTGTGGT
	R AGTGTTGATGTCAGTGTGAGC
Il-10	F GTCATCGATTTCTCCCCTGTG
	R ATGGCCTTGTAGACACCTTG
Tnf-α	F AGACCCTCACACTCAGATCA
	R TCTTTGAGATCCATGCCGTTG

**Table 3:** Body composition and bone mineral density.

	Dietary Groups	Baseline	6 Months	12 Months
Lean mass (g)	Control	22.63 ± 0.55	25.88 ± 0.65 ^ab^	26.45 ± 0.63 ^a^
	Control + BCAA	22.30 ± 0.48	26.33 ± 0.77 ^ab^	26.45 ± 0.79 ^a^
	Low protein	22.37 ± 0.43	23.67 ± 0.58 ^b^	23.61 ± 0.48 ^b^
	Low protein + BCAA	22.86 ± 0.53	26.49 ± 0.52 ^a^	27.04 ± 0.63 ^a^
Fat mass (g)	Control	4.33 ± 0.16	11.10 ± 0.56 ^ab^	12.67 ± 0.67 ^ab^
	Control + BCAA	4.37 ± 0.14	11.82 ± 0.70 ^ab^	14.21 ± 0.75 ^ab^
	Low protein	4.51 ± 0.21	9.14 ± 0.88 ^b^	10.73 ± 0.94 ^b^
	Low protein + BCAA	4.22 ± 0.16	12.98 ± 0.59 ^a^	15.95 ± 0.88 ^a^
Femur BMD (g/cm^2^)	Control	0.071 ± 0.002	0.079 ± 0.001	0.076 ± 0.002
	Control + BCAA	0.067 ± 0.002	0.075 ± 0.002	0.074 ± 0.001
	Low protein	0.067 ± 0.002 *	0.073 ± 0.001 *	0.071 ± 0.001 *
	Low protein + BCAA	0.072 ± 0.002	0.076 ± 0.002	0.073 ± 0.001
Femur BMC (g)	Control	0.037 ± 0.002	0.038 ± 0.001	0.036 ± 0.001
	Control + BCAA	0.037 ± 0.002	0.036 ± 0.001	0.034 ± 0.001
	Low protein	0.034 ± 0.001	0.034 ± 0.001	0.031 ± 0.001
	Low protein + BCAA	0.037 ± 0.001	0.035 ± 0.001	0.033 ± 0.001

Data represent mean ± standard error of the mean (SEM) (*n* = 17–20 mice/group). Different letters (a, b) denote significant differences between groups in Tukey multiple comparisons test within the same time point. * *p* < 0.05 for control vs. low protein. BMD: bone mineral density; BMC: bone mineral content.

**Table 4:** Top canonical pathways identified in liver proteomics analysis.

Dietary Groups	Name	Overlap	Molecules (Direction of Regulation)
Low protein vs. Control	Oxidative phosphorylation	18/109	COX4I1, NDUFA12, SDHA, NDUFB8, NDUFV1, NDUFA2, ATP5D, COX6C, NDUFS1, SDHB, COX7A2, NDUFS2, ATP5L, NDUFA6, NDUFA3, ATP5C1, NDUFB4, NDUFV2 (all upregulated by low protein)
	Mitochondrial dysfunction	19/171	COX4I1, HSD17B10, NDUFA12, SDHA, NDUFB8, NDUFV1, NDUFA2, ATP5D, COX6C, NDUFS1, SDHB, COX7A2, NDUFS2, ATP5L, NDUFA6, NDUFA3, ATP5C1, NDUFB4, NDUFV2 (all upregulated by low protein)
	Superpathway of Citrulline Metabolism	8/14	OAT, OTC, CPS1, ASS1, ARG1, PRODH, GLS2, ASL (all upregulated by low protein)
	Urea Cycle	5/6	OAT, CPS1, ASS1, ARG1, ASL (all upregulated by low protein)
	Citrulline Biosynthesis	5/8	OAT, OTC, ARG1, PRODH, GLS2 (all upregulated by low protein)
Low protein vs. Low protein + BCAA	Tyrosine Degradation I	3/5	FAH, HGD, HPD (all downregulated by low protein)
	Urea Cycle	3/6	OTC, ARG1, ASL (all downregulated by low protein)
	Cysteine Biosynthesis/Homocysteine Degradation	2/2	CBS, CTH (both downregulated by low protein)
	Superpathway of Citrulline Metabolism	3/14	OTC, ARG1, ASL (all downregulated by low protein)
	TCA Cycle II (Eukaryotic)	3/23	SDHB, FH, SDHA (all downregulated by low protein)

Pathways identified by INGENUITY when comparing different dietary groups. *n* = 8 mice per group. See Table S2 for full protein names.

## Supplementary Materials

The following are available at http://www.mdpi.com/2072-6643/10/7/918/s1, Table S1: Statistical analysis of body composition and bone mineral density data, Figure S1: Representative FACS density plots demonstrating the overall CD4^−^CD8^−^, CD4^+^CD8^+^, CD4^+^ and CD8^+^ populations of thymocytes after the dietary intervention, Figure S2: Representative FACS density plots demonstrating the overall CD4^+^ and CD8^+^ populations after the dietary intervention, Table S2: Protein abbreviations.
