# Supplementary material for: Long-Term Effects of Dietary Protein and Branched-Chain Amino Acids on Metabolism and Inflammation in Mice
Source: Nutrients. 2018 Jul 18;10(7):918. doi: 10.3390/nu10070918 (PMC6073443; doi:10.3390/nu10070918)
Supplement: Supplementary file 1 [file nutrients-10-00918-s001.pdf]

Supplementary Materials: Long-Term Effects of Dietary Protein and Branched-Chain Amino Acids on Metabolism and Inflammation in Mice

Wei-Chieh Mu [1], Erin VanHoosier [1], Carrie M. Elks [2], and Ryan W. Grant [1,*]

Table S1. Statistical analysis of body composition and bone mineral density data.

	Protein	BCAA	Protein* BCAA	Time	Protein* BCAA* Time
Lean mass	$p = 0.2741$	$p = 0.0254$	$p = 0.0312$	$p < 0.0001$	$p = 0.0483$
Fat mass	$p = 0.7838$	$p = 0.0004$	$p = 0.0325$	$p < 0.0001$	$p = 0.0245$
Femur BMD	$p = 0.2078$	$p = 0.9265$	$p = 0.0143$	$p < 0.0001$	$p = 0.3251$
Femur BMC	$p = 0.0571$	$p = 0.9753$	$p = 0.1338$	$p < 0.0001$	$p = 0.9527$

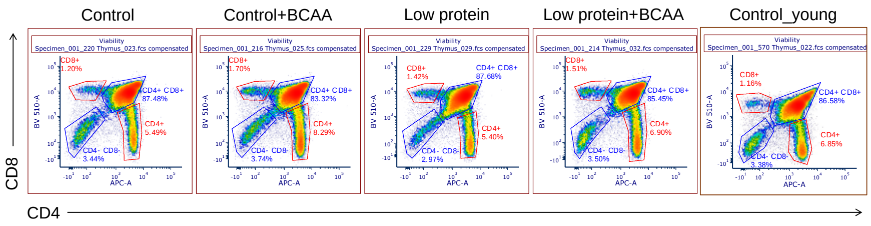

Figure S1. Representative FACS density plots demonstrating the overall $CD4^-CD8^-$, $CD4^+CD8^+$, $CD4^+$ and $CD8^+$ populations of thymocytes after the dietary intervention.

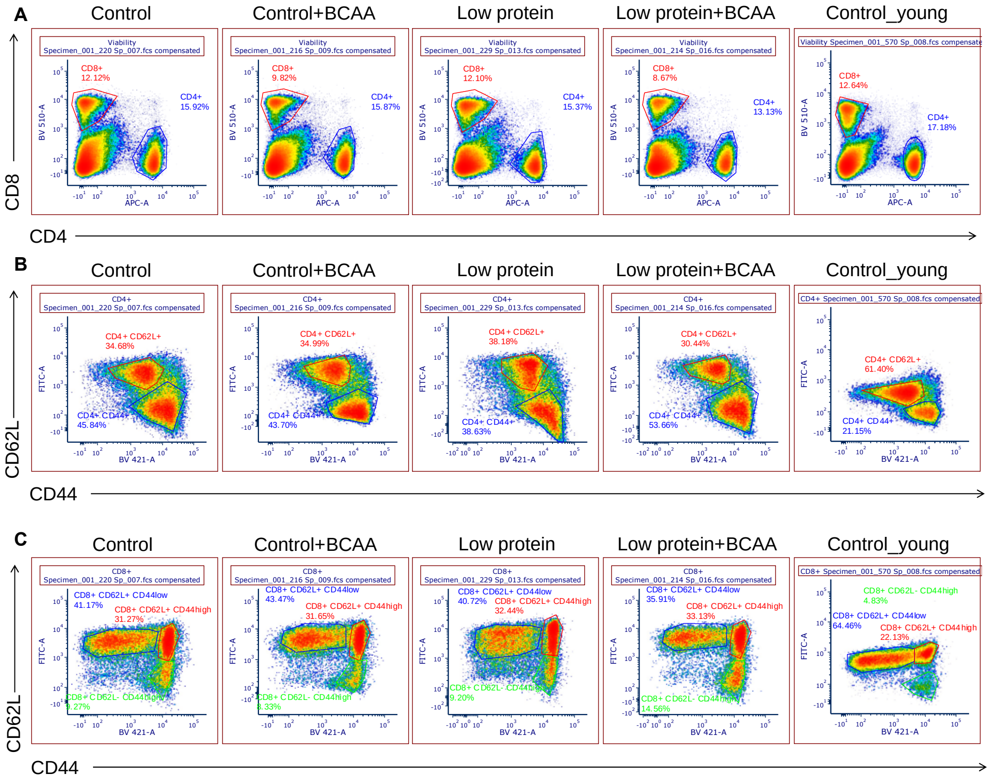

Figure S2. Representative FACS density plots demonstrating the overall $CD4^+$ and $CD8^+$ populations after the dietary intervention (A). Representative FACS density plots demonstrating $CD4^+$ T-cell subpopulations; naïve ($CD4^+CD62L^+CD44^-$) and memory ($CD4^+CD62L^-CD44^+$) populations (B). Representative FACS density plots demonstrating $CD8^+$ T-cell subpopulations; naïve

($CD8^+CD62L^+CD44^-$), central memory ($CD8^+CD62L^+CD44^+$) and effector memory ($CD8^+CD62L^-CD44^+$) populations (C).

Table S2. Protein abbreviations.

Abbreviation	Name
ARG1	Arginase 1
ASL	Argininosuccinate lyase
ASS1	Argininosuccinate synthase 1
ATP5C1	ATP synthase membrane subunit G
ATP5D	ATP synthase F1 subunit delta
ATP5L	ATP synthase F1 subunit gamma
CBS	Cystathionine-beta-synthase
COX4I1	Cytochrome C oxidase subunit 4I1
COX6C	Cytochrome C oxidase subunit 6C
COX7A2	Cytochrome C oxidase subunit 7A2
CPS1	Carbamoyl-phosphate synthase 1
CTH	Cystathionine gamma-lyase
FAH	Fumarylacetoacetate hydrolase
FH	Fumurate hydratase
GLS2	Glutaminase 2
HGD	Homogentisate 1,2-dioxygenase
HPD	4-hydroxyphenypyruvate dioxygenase
HSD17B10	Hydroxysteroid 17-beta dehydrogenase 10
NDUFA12	NADH:ubiquinone oxidoreductase subunit A12
NDUFA2	NADH:ubiquinone oxidoreductase subunit A2
NDUFA3	NADH:ubiquinone oxidoreductase subunit A3
NDUFA6	NADH:ubiquinone oxidoreductase subunit A6
NDUFB4	NADH:ubiquinone oxidoreductase subunit B4
NDUFB8	NADH:ubiquinone oxidoreductase subunit B8
NDUFS1	NADH:ubiquinone oxidoreductase subunit S1
NDUFS2	NADH:ubiquinone oxidoreductase subunit S2
NDUFV1	NADH:ubiquinone oxidoreductase subunit V1
NDUFV2	NADH:ubiquinone oxidoreductase subunit V2
OAT	Ornithine aminotransferase
OTC	Ornithine carbamoyltransferase
PRODH	Proline dehydrogenase 1
SDHA	Succinate dehydrogenase complex flavoprotein subunit A
SDHB	Succinate dehydrogenase complex iron sulfur subunit B
